# Scalp Eschar and Neck Lymphadenopathy Caused by *Rickettsia massiliae*

**DOI:** 10.3201/eid1905.121169

**Published:** 2013-05

**Authors:** Antonio Cascio, Alessandra Torina, Mariella Valenzise, Valeria Blanda, Natalia Camarda, Sara Bombaci, Chiara Iaria, Filippo De Luca, Malgorzata Wasniewska

**Affiliations:** University of Messina, Messina, Italy (A. Cascio, A. Torina, M. Valenzise, N. Camarda, S. Bombaci, F. De Luca, M. Wasniewska);; Istituto Zooprofilattico Sperimentale della Sicilia, Palermo, Italy (A. Torina, V. Blanda);; Azienda Ospedaliera Piemonte-Papardo, Messina (C. Iaria)

**Keywords:** Rickettsia, lymphadenopathy, fever, tick, bacteria, eschar, scalp eschar and neck lymphadenopathy, Rickettsia massiliae

**To the Editor:** Scalp eschar and neck lymphadenopathy is a common clinical entity that most frequently affects women and children during spring and fall. It is usually caused by *Rickettsia slovaca* and *R. raoultii*. Typical clinical signs are a scalp lesion at the tick bite site and regional, often painful, lymphadenopathy. Acute disease can be followed by residual alopecia at the bite site (*1*,*2*). Two designations have been proposed for this syndrome: tick-borne lymphadenopathy and *Dermacentor*-borne necrosis-erythema-lymphadenopathy (both have been associated with *R. slovaca*); however, the most generic and all-inclusive term is scalp eschar and neck lymphadenopathy.

*R. massiliae* belongs to the spotted fever group rickettsiae, is distributed worldwide, and is transmitted by ticks of the genus *Rhipicephalus* (*3*). To our knowledge, only 3 cases of *R. massiliae* infection in humans have been documented and confirmed by molecular methods. The first case was detected in a blood sample from a patient in Italy who had Mediterranean spotted fever (*4*); the second case was in a patient in southern France who had spotted fever and acute loss of vision (*5*); and the third case was in a woman in Argentina who had fever, a palpable purpuric rash, and tache noire (*3*). We report a case of *R. massiliae* infection that resulted in scalp eschar and neck lymphadenopathy*.*

 On May, 10, 2012, a 13-year-old boy was examined for headache, high fever, and right painful neck and occipital swelling. Six days earlier, a tick had been removed from the top of his scalp, after which signs and symptoms arose and gradually worsened.

Physical examination revealed temperature 39.5°C, pulse rate 70 beats/min, and respiratory rate 20 breaths/min. The boy appeared to be in good condition. An ≈1-cm black eschar was noted at the site of the tick bite. Palpation of the neck revealed painful bilateral adenopathies. Other lymph nodes in the occipital region were enlarged. No exanthema was noted, the liver was palpable 1 cm under the costal margins, and the spleen was not enlarged. Laboratory evaluation indicated blood cell counts and liver and kidney function within reference limits, mild elevation of inflammatory markers (C-reactive protein 1.2 mg/dL [reference <0.5 mg/dL]), and elevated erythrocyte sedimentation rate (43 mm/h). Ultrasonography of the neck confirmed the presence of numerous, enlarged, oval lymph nodes (maximum 17 mm) with hilar vascularity within normal limits. A scalp eschar biopsy sample and acute- and convalescent-phase (day 30) serum samples were sent to the Istituto Zooprofilattico Sperimentale della Sicilia.

The patient was given doxycycline at 100 mg 2 times per day. Signs and symptoms began to improve 48–72 h later and gradually disappeared. Fever was gone after 3 days, and the other symptoms had regressed after 7 days.

Serologic testing for *R. conorii* was performed by microimmunofluorescence with the *R. conorii/R. typhi* IgG MIF Kit (Fuller Laboratories, Fullerton, CA, USA). Total DNA was extracted from blood and the eschar by GenElute Mammalian Genomic DNA Miniprep (Sigma-Aldrich, St. Louis, MO, USA). To detect *Rickettsia* spp. DNA, we tested nucleic acids by PCR with a set of primers that amplify a 256-bp region of the gene encoding the 17-kDa antigen (*6*). To obtain information about *Rickettsia* spp., we amplified regions of the genes *gltA* (*7*,*8*), *ompA* (*7*), and *ompB* (*9*). PCR products were purified by the Wizard SV Gel and PCR Clean-up System (Promega, Madison, WI, USA), quantified, and sent for sequencing to Macrogen Inc. (Amsterdam, the Netherlands).

Obtained sequences were aligned and analyzed by using Bioedit software (Ibis Biosciences, Carlsbad, CA, USA) and ClustalW version 2.0.10 (www.ebi.ac.uk/clustalw). DAMBE (http://dambe.bio.uottawa.ca/dambe.asp) and MEGA (www.megasoftware.net) software were used to obtain similarity percentages among analyzed sequences. To characterize *Rickettsia* spp., we used nucleotide sequence identity to reference strains (*10*).

Convalescent-phase serum was positive for *R. conorii*; IgG titer was 64. Sequence analysis of purified PCR products obtained from the eschar identified the isolate as *R. massiliae*. With respect to the reference strain *R. massiliae,* pairwise nucleotide sequence identity was 99% for the *gltA* gene (GenBank accession no. JN043507), 99% for the *ompA* gene (accession no. JQ480842), and 97% for the *ompB* gene (accession no. AF123714). Phylogenetic analysis (Technical Appendix) also confirmed the identity of the *Rickettsia* species.

Considering the diagnosis of *R. massiliae* infection and the patient who had acute vision loss (*5*), this patient was called back for a fundus examination, which showed no changes. At the time of this visit, a small area of alopecia at the eschar site was observed (Figure). Unfortunately, the tick had been discarded and was not available for genus and species identification.

**Figure:**
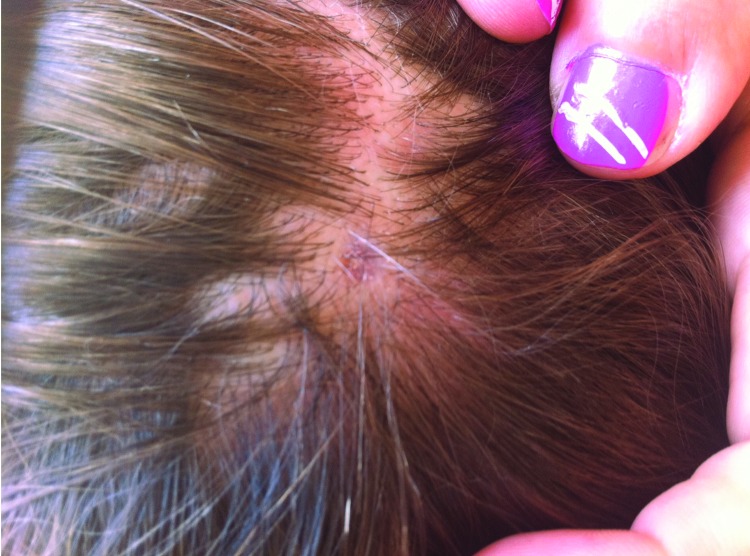
Residual alopecia 10 weeks after tick bite in 13-year-old boy with scalp eschar and neck lymphadenopathy caused by *Rickettsia massiliae*. Printed with permission from N.C. (photographer and author) and from parents of the patient.

The presence of *R. massiliae* in Italy demonstrates that this *Rickettsia* species can cause scalp eschar and neck lymphadenopathy. Further studies are needed to complete the list of microorganisms that can cause this condition and to understand if they can be associated with minor findings (e.g., alopecia, painful eschar, high fever).

Technical Appendix Phylogenetic analysis of *Rickettsia* spp.
