# Supplementary material for: Scalp Eschar and Neck Lymphadenopathy Caused by Rickettsia massiliae
Source: Emerg Infect Dis. 2013 May;19(5):836. doi: 10.3201/eid1905.121169 (PMC3647502; doi:10.3201/eid1905.121169)
Supplement: Technical Appendix — Phylogenetic analysis of Rickettsia spp. [file 12-1169-Techapp-s1.pdf]

Article DOI: http://dx.doi.org/10.3201/eid1905.121169

Scalp Eschar and Neck Lymphadenopathy Caused by *Rickettsia massiliae*

Technical Appendix

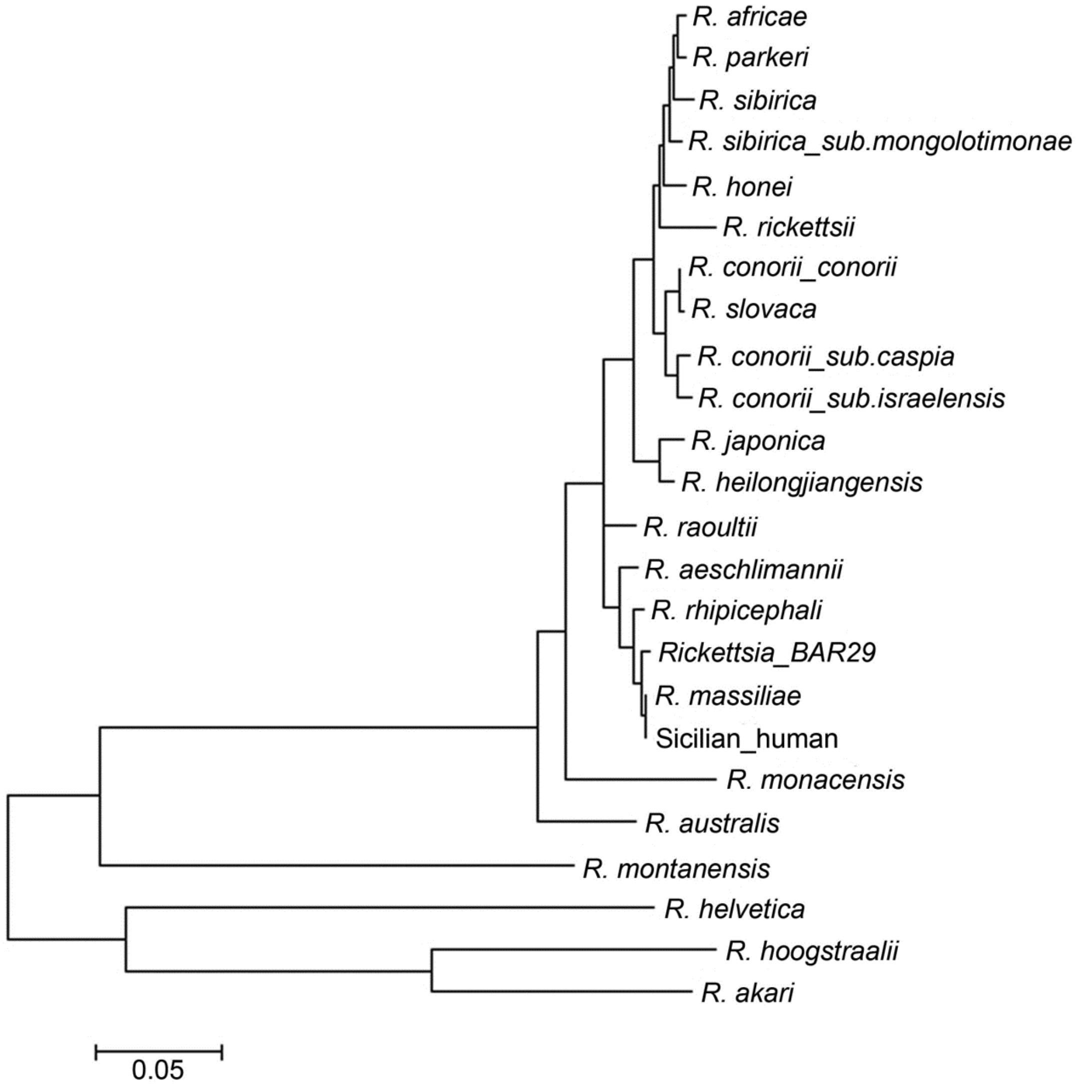

Technical Appendix Figure. Phylogenetic analysis of *Rickettsia* spp. Evolutionary history was inferred by using the neighbor-joining method for *ompA* gene, *ompB* gene, and *gltA* gene. “Human” indicates 13-year-old boy with scalp eschar and neck lymphadenopathy caused by *Rickettsia massiliae*.
